# Non-synonymous variations in cancer and their effects on the human proteome: workflow for NGS data biocuration and proteome-wide analysis of TCGA data

**DOI:** 10.1186/1471-2105-15-28

**Published:** 2014-01-27

**Authors:** Charles Cole, Konstantinos Krampis, Konstantinos Karagiannis, Jonas S Almeida, William J Faison, Mona Motwani, Quan Wan, Anton Golikov, Yang Pan, Vahan Simonyan, Raja Mazumder

**Affiliations:** 1 Department of Biochemistry and Molecular Medicine, George Washington University Medical Center, Washington, DC 20037, USA; 2 J. Craig Venter Institute, 9704 Medical Center Drive, Rockville, MD 20850, USA; 3 Division of Informatics of the Department of Pathology, University of Alabama at Birmingham, Birmingham, AL 35294, USA; 4 Center for Biologics Evaluation and Research, US Food and Drug Administration, 1451 Rockville Pike, HFM-470, Rockville, MD 20852, USA; 5 McCormick Genomic and Proteomic Center, George Washington University, Washington, DC 20037, USA

**Keywords:** SRA, TCGA, nsSNV, SNV, SNP, Next-gen, NGS, Phylogenetics, Cancer

## Abstract

**Background:**

Next-generation sequencing (NGS) technologies have resulted in petabytes of scattered data, decentralized in archives, databases and sometimes in isolated hard-disks which are inaccessible for browsing and analysis. It is expected that curated secondary databases will help organize some of this Big Data thereby allowing users better navigate, search and compute on it.

**Results:**

To address the above challenge, we have implemented a NGS biocuration workflow and are analyzing short read sequences and associated metadata from cancer patients to better understand the human variome. Curation of variation and other related information from control (normal tissue) and case (tumor) samples will provide comprehensive background information that can be used in genomic medicine research and application studies. Our approach includes a CloudBioLinux Virtual Machine which is used upstream of an integrated High-performance Integrated Virtual Environment (HIVE) that encapsulates Curated Short Read archive (CSR) and a proteome-wide variation effect analysis tool (SNVDis). As a proof-of-concept, we have curated and analyzed control and case breast cancer datasets from the NCI cancer genomics program - The Cancer Genome Atlas (TCGA). Our efforts include reviewing and recording in CSR available clinical information on patients, mapping of the reads to the reference followed by identification of non-synonymous Single Nucleotide Variations (nsSNVs) and integrating the data with tools that allow analysis of effect nsSNVs on the human proteome. Furthermore, we have also developed a novel phylogenetic analysis algorithm that uses SNV positions and can be used to classify the patient population. The workflow described here lays the foundation for analysis of short read sequence data to identify rare and novel SNVs that are not present in dbSNP and therefore provides a more comprehensive understanding of the human variome. Variation results for single genes as well as the entire study are available from the CSR website (http://hive.biochemistry.gwu.edu/dna.cgi?cmd=csr).

**Conclusions:**

Availability of thousands of sequenced samples from patients provides a rich repository of sequence information that can be utilized to identify individual level SNVs and their effect on the human proteome beyond what the dbSNP database provides.

## Background

Researchers in cancer biology are well aware of the possibilities that Big Data can offer and there are already several efforts underway to generate relevant large-scale cancer genomics data [1-3]. International and national networks of collaboration, such as International Cancer Genome Consortium (ICGC) [4], Global Cancer Genomics Consortium (GCGC) [5], Early Detection Research Network (EDRN) [6] and other NCI programs (like The Cancer Genome Atlas program (TCGA) [7]), are generating increasingly large amounts of data, the vast majority of which is from next-generation sequencing (NGS) technologies. TCGA data is expected to surpass 100 petabytes by the completion of the project. In a recent survey of the files generated by the TCGA initiative we found that its file count has been doubling every 7 months since 2010, with a total count above 700,000 files. Although, it is desirable that cancer biologists will use this data to develop and test hypotheses, realistically, few wet-laboratory researchers have the infrastructure or knowledge regarding scores of complex bioinformatics tools to glean a higher understanding from the disparate sequence files and complex, scattered annotations. These challenges are leading to the development of tools and secondary databases which are expected to democratize Big Data use [8-10], and initiatives such as the Human Variome Project has started playing an important role by providing guidelines that encourage standardizing and sharing of information related to human genetic variation [11-13].

Biological information is usually concentrated in databases mainly of two types: primary databases comprised of raw data submitted by researchers, and secondary databases that extract and filter the information available from the primary databases and add additional annotations generated either manually or automatically through the efforts of biocurators [14-16]. One of the problems often faced by end users of Big Data is the lack of curated information in primary NGS data repositories such as the NCBI Short Read Archive (NCBI-SRA) [17] and The Cancer Genomics Hub (CGHub) [18]. It is expected that curated secondary databases will help organize the Big Data and make it more user-friendly, similar to what secondary database development efforts like RefSeq [19] and UniProtKB/Swiss-Prot [20] have done and are still doing for GenBank [21]. Additional higher level databases like Pfam [22], PIRSFs [23], KEGG [24] and others organize objects into functional groups and provide information on biological function, networks and processes. It is clear that knowledge can be gained when raw data moves in a vertical fashion, from millions of bases of DNA or RNA into proteins, then to protein families, and finally into networks of interrelated biological processes. Currently, NGS data in public repositories are not well connected to molecular biology resources and reference datasets, and validated methods for data processing and filtering are not always available, necessitating significant bioinformatics expertise to use and analyze such information. In this paper, we describe a workflow to curate and analyze NGS data from control (normal tissue) and case (tumor) samples derived from cancer patients. We chose to curate publicly available NGS data to provide users an unbiased view of variation that is present at the individual person level and is not yet completely captured in dbSNP, thereby providing a better understanding of the human variome. Additionally, based on our previous work on functional analysis of non-synonymous Single Nucleotide Variations (nsSNVs) from dbSNP [21], UniProt [20] and COSMIC [25] we show how proteome-wide analysis of variation can provide a detailed view of the distribution of variation and possible functional impact [26-29].

Currently, there are thousands of large-scale sequence data from cancer case and control samples that are available from primary short read data repositories such as NCBI SRA and TCGA. It is expected that comprehensive and integrated analysis of this data will lead to novel discoveries. We believe that computational and manual curation of this data will provide unprecedented value in cancer research that will eventually lead to better cancer detection, therapies, and care. As proof-of-concept, we have analyzed nsSNVs from 55 samples (22 cases and 33 controls) obtained from 20 breast cancer patients and have recorded the analysis results in Curated Short Read (CSR) archive. The samples provide a rich source of sequence data that can be mined to extend and compliment mutation and single-nucleotide polymorphism (SNP) information available from dbSNP [21], UniProt [20], COSMIC [25] and other variation databases. We intend to curate and analyze representative samples from all datasets that are available through TCGA. For our initial study, through focused analysis of the breast cancer samples, we show how a workflow that identifies novel variations, explores the effects of nsSNVs on the human proteome and classification of patients based on Single Nucleotide Variations (SNVs) can provide a higher level of information that can be used by researchers to evaluate experimental targets and also to generate and test hypothesis related to personalized medicine. To facilitate implementation of this workflow by other users, we provide nsSNV analysis tool - SNVDis that can be used by researchers or biocurators interested in evaluating the effects of variation. With large-scale informatics fast becoming an integral component of cancer research, the workflow described here can be easily applied to other datasets.

## Methods

### Architecture and computational environment

**Figure 1:**
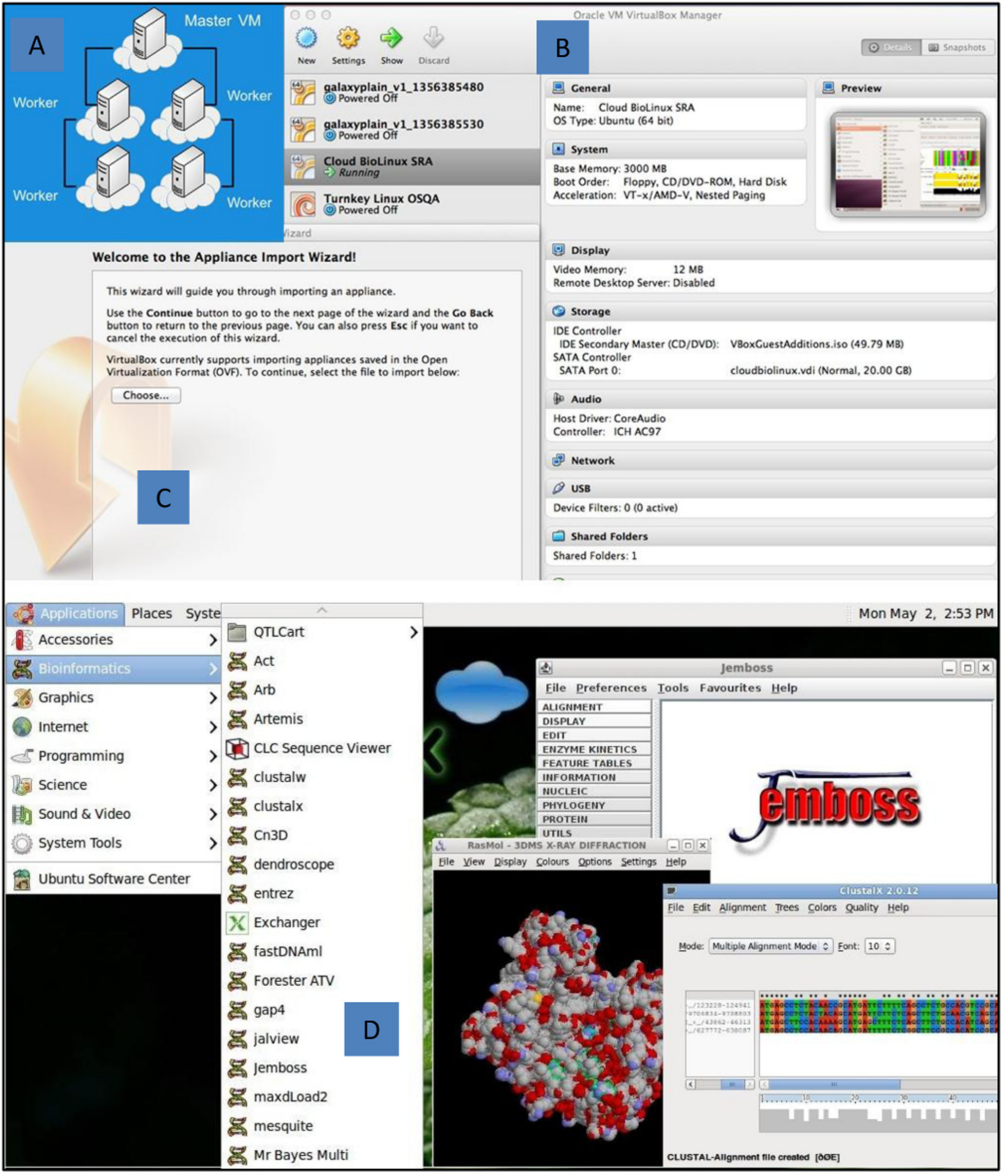
**Cloud BioLinux environment. A)** Computational infrastructure; **B)** Cloud BioLinux SRA provides a command line interface for mapping and SNV identification and Oracle VM VirtualBox manager allows user to edit settings **C)** Appliance Import Wizard allows import of appliance in Open Virtualization format. **D)** Snapshot of applications available through Cloud BioLinux environment.

All data displayed on the website as well as any data or references used in the analysis are stored in the High-performance Integrated Virtual Environment (HIVE) server (http://hive.biochemistry.gwu.edu) [30]. The results are searchable and are also available as tab-delimited files. Users can either browse the curated data or search for specific genes or proteins using RefSeq accession number as the query. Searches with TCGA IDs and UniProtKB accession numbers are also supported. HIVE provides the storage and analysis computational infrastructure for this project. Resources include six Dell Servers, an integrated computational environment, and 10GB 48port Ethernet switch and control servers. The workflow is also incorporated within the CloudBioLinux [31] environment (Figure 1). Cloud BioLinux VM can be downloaded from https://s3.amazonaws.com/cloudbiolinuxvms/cloudbiolinuxsra/cloudbiolinuxsra.ova. Installation instructions are available at http://www.cloudbiolinux.org and http://www.virtualbox.org/manual/ch01.html#ovf.

### Datasets

Short read data is obtained from The Cancer Genome Atlas (http://cancergenome.nih.gov/) via the CGHub data portal (https://cghub.ucsc.edu/). The reference used in the alignment is the hg19,

GRCh37 Genome Reference Consortium Human Reference 37 (GCA_000001405.1) downloaded from UCSC (http://hgdownload.cse.ucsc.edu/goldenPath/hg19/chromosomes/). UniProtKB protein amino acid position and ID mapping is done using SNVDis and ID Mapping services [28,32]. Functional and sequence information is obtained from RefSeq [19], Conserved Domain Database [33], UniProtKB/Swiss-Prot [20] and CCDS [34].

### Data selection criteria

For this study, we concentrate on sequence data from breast cancer cases and controls. Data sets derived from twenty patients are selected for analysis. The criterion for selection is based on the availability of clinical information, race, paired case and control samples. In addition to this, the presence of both exome and RNA-Seq data from the same patient is also included as a criterion for selection because they are deemed to be high-priority datasets by our users because many hypothesis driven questions can be answered through the comparative analysis of these datasets. Information is retrieved using the data matrix available at https://tcga-data.nci.nih.gov/tcga/dataAccessMatrix.htm. “BRCA-Breast invasive carcinoma” is selected as “Disease type” and “Clinical” from the “Data Type”. Patients from three different races are included: three patients from African-American, one Asian, and the rest White. All patients are females and had no previous history of malignancy. The tumor and the matched control samples can be identified by the TCGA barcode associated with each sample. A TCGA barcode is a collection of identifiers such as in the sample ID (TCGA-CH-5739-01A-11D-1576-08), where 5739 is the participant number, 01 is the sample type. Tumor sample range from 01–09 and matched normal from 10 – 19 depending on the type of tumor and normal sample. The short reads are then mapped to the human reference sequence and further analyzed to identify variations.

### SNV filtration and annotation

After the raw SNV data is generated using Bowtie [35] and SAMtools [36], filters are used to select high quality SNVs which are of desirable coverage (>10 reads) and quality score (>20). The filtration process also rejects detected SNVs falling out of the exome regions, which may be caused by non-unique regions in the genome.

### nsSNV distribution on functional sites

Experimental Post Translational Modification (PTM) sites are obtained from UniProtKB/Swiss-Prot [20] and dbPTM 3.0 [37], which provides experimentally verified PTM sites. Python scripts are used to remove the redundancy from the nsSNVs dataset, map the resulting unique nsSNVs to PTM motifs in UniProtKB proteins, and calculate how many PTM sites are detected to have nsSNV. Any nsSNV derived unacceptable change on the given PTM motif is considered as a loss of PTM site. To evaluate the effects of nsSNVs obtained from case and control samples, statistics were generated and heatmap was constructed using R package [38]. To obtain the heatmap, the binary matrix data of presence or absence of SNV in a particular position is loaded into R and using the packages ggplot2 and reshape, the binary heatmap is obtained by converting the values of each cell into either red ‘present in only case or control’ or green ‘present in both case and control’. This information is then plotted with the amino acid positions across the vertical axis.

### Tools

The CGquery and Gene Torrent utilities (https://cghub.ucsc.edu/software/downloads.html) are used to search for and retrieve BAM files from CGHub. Alignment is performed using Bowtie version 0.12.5 [35]. SNVs are calculated using SAMtools version 0.1.18 and bcftools version 0.1.17 [36]. The pipeline consists of a series of Perl scripts and the above-mentioned software which are called using a wrapper script. The wrapper is accessed via the command line and accepts two arguments, a list of fastq files that needs to be analyzed and the location where the output should be placed. The statistics are generated with R [38].

### Phylogenetic analysis and SNV visualization

A novel algorithm (phyloSNP) has been developed to create SNV-based phylogentic trees. The first step involves creating an alignment that contains genomic sequence around SNVs. For this study we chose to include zero, one and two nucleotides upstream and downstream of every SNV to create SNV-shrunk genome. More specifically, the SNV-shrunk genome alignments are created using phyloSNP (https://hive.biochemistry.gwu.edu/hive/dna.cgi?cmd=phylosnp) by concatenating regions of the genome that has SNV for each sample. If one sample has a SNV in a particular position then all of the other SNV-shrunk genomes from the other samples include that region in their SNV-shrunk genome. Therefore the output of all SNV-shrunk genomes is an alignment. This alignment is then used to generate neighbor joining phylogenetic trees using Clustal with 100 bootstrap values [39]. Bootstrap values indicate the confidence of the branches in the estimated trees. The trees are viewed in TreeView [40].

### Functional analysis

Filtered SNVs are submitted to Seattleseq 137 Annotation Service [41] to get positional and functional annotation. Additional functional analysis of proteins affected by nsSNVs is performed based on methods described earlier [26-28]. Briefly, nsSNV data is uploaded into SNVDis database and integrated with protein sequence features obtained from UniProtKB/Swiss-Prot [20], Conserved Domain Database (CDD) and RefSeq of NCBI [42]. SNVDis provides graphical and tabular output of variations that affect functionally annotated sequence sites. Additionally, SNVDis also provides information if there is an over- or under-representation of certain pathways and domains that are affected by nsSNVs. Gene Ontology analysis of genes affected by rare variants is performed using PANTHER tools [43,44].

### Key definitions

Case and control – Samples derived from paired tumor (case) and normal (control) tissue; dbSNP overlap – SNVs that are also found in dbSNP; novel SNV – found only in the analyzed dataset; rare SNV – found in less than 10% of the samples analyzed; common SNV – found in 90% of the samples analyzed.

## Results and discussion

SNVs are widely used to identify disease causing genes and history of populations [45-47]. Many advances in the diagnosis and treatment of cancers have been made through such mutation discovery and analysis [48-52]. Combining the results of several studies (meta-analysis) can increase the power of the analysis [53]. These meta-analyses combine the results (SNVs) from multiple studies and, using different statistical tools, identify the SNVs most associated with a specific disease or phenotype. Analysis of samples across different studies would provide a glimpse of the heterogeneity that is present in the population and this information can then be used by researchers to connect genomic changes to diseases. Additionally, availability of variation data from control samples can provide a more comprehensive understanding of the human variome in addition to what has been determined by projects such as the 1000 Genomes project [29]. The effects that a specific variation has on a protein function have been the focus of studies for quite some time with several tools that predict SNV effects [54-57]. Proteome-wide analysis of variation that affects known functional sites [26-28,58] is another way of estimating how variation can affect function at a system level and if there are specific domains or pathways that are more prone to having variations.

**Figure 2:**
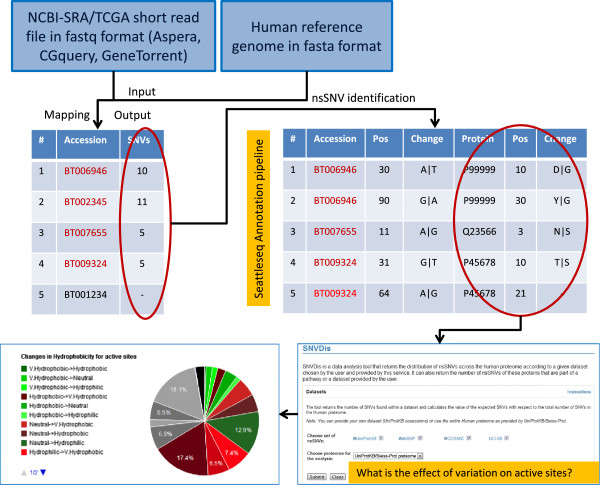
**Short read sequence mapping and nsSNV analysis workflow.** nsSNV variations are mapped to proteins to identify amino acid changes. Functional site-specific information is extracted from UniProtKB, RefSeq and Conserved Domain Database.

In this study, we analyze patient derived samples to identify nsSNVs that are in concordance with dbSNP and also novel variations, determine the effect of nsSNVs on the proteome and classify the patients based on phylogentic analysis of the nsSNVs. Figure 2 provides a flowchart of the workflow that involves retrieving sequence data from TCGA followed by mapping the short sequence reads to human reference genome and identification of SNVs. Subsequently, SNVs are mapped to protein sequences and the proteome-wide distribution of SNV is investigated. All of the nsSNV data and sample-specific annotations are then recorded and is made available at the Curated Short-Read database (CSR). Users studying specific cancer genes or proteins or the entire patient proteome can query CSR in addition to known variation databases such as dbSNP, COSMIC and UniProt to get a comprehensive view of variation. Additionally, because all of the data from this study, dbSNP, COSMIC and UniProt are integrated into SNVDis, users can evaluate the effects of all variations in addition to browsing them in CSR.

### Metadata curation

There is a great need in biological research and discovery for curated metadata that is associated with short sequence reads. Just like GenBank, NCBI-SRA and other public repository of short sequence reads around the world are all primary databases with minimal or no curation. This means that it is extremely difficult for users to search for and retrieve studies that can be used for additional analysis or browse analysis results that are associated with specific genes of interest.

Sequencing has identified key disease specific mutations in many cancers where the authors filter variation information from dbSNP to identify cancer specific variations [59]. The data in dbSNP does not yet capture all possible individual level variation. Hence we intend to focus on analyzing and curating samples which can be used in conjunction with dbSNP data to better understand the human variome. For comparison purposes both cases and controls are analyzed. The key fields that we focus our curation efforts on are as follows: 1) Study, experiment and sample title, type, abstract and associated publications; 2) Organism name and taxonomy ID; 3) Additional information wherever applicable such as sample type, tissue site, clinical status, age, gender, ethnicity and gleason score; 4) Identification of nsSNVs; 5) Mapping of nsSNVs to dbSNP. In this project for tasks one, two and three data is obtained from TCGA files and manually verified. Publications that use TCGA data files are searched for in PubMed [60] and manually checked to confirm that they report analysis of cancer specific data that is under consideration. For tasks four and five a computational approach that involves read mapping and SNV calling followed by spot checks is performed. All metadata data in CSR is manually verified and entered. Samples which do not have the acceptable GC content of between 38-48% are not processed for curation.

The scientific name of the organism and the taxonomy id are based on NCBI Taxonomy which are *Homo sapiens* and 9606 respectively. The analysis presented here includes 55 samples from 20 patients where experiment numbers CA00001BC - CA00022BC belong to cases and CO00001BC - CO00033BC to controls. Case and control samples start with a prefix CA and CO respectively, followed by numbers and ends with a prefix for the cancer type (Breast Cancer – BC). Each experiment accession number is associated with a unique sample accession number and belongs to same study. The experiment contains information regarding sequencing library strategy, source, selection, layout and platform, which are obtained from the metadata at CGhub. The CSR database provides easy access to gene specific nsSNV variations found in specific samples and also downloads of nsSNVs of case and control samples with mappings to dbSNP which can be used for additional analysis purposes. Table 1 provides a snapshot of the information obtained when a protein or gene accession number is used to search the CSR database. CSR data is also integrated into SNVDis for proteome-wide analysis as described in the functional analysis section below.

**Table 1:** Snapshot of information obtained upon searching the CSR database with a protein or gene accession number

**RefSeq nucleotide AC**	**Pos**^**1**^	**Chg**^**2**^	**Protein**	**Pos**^**3**^	**Chg**	**UniProt AC**^**4**^	**Pos**^**5**^	**Sample**	**Type**	**Library**
NM_000059.3	1092	A|C	NP_000050.2	289	N|H	P51587	289	TCGA-BH-A0BW-01A-11D-A10Y-09	Breast Cancer Case	WXS^6^
NM_000059.3	1205	C|A	NP_000050.2	326	S|R	P51587	326	TCGA-BH-A0AZ-01A-21D-A12Q-09	Breast Cancer Case	WXS
NM_000059.3	1341	A|C	NP_000050.2	372	N|H	P51587	372	TCGA-AC-A2FF-01A-11D-A17D-09	Breast Cancer Case	WXS

^1^Position in RefSeq nucleotide entry.

^2^Nucleotide change.

^3^Position in RefSeq protein entry.

^4^UniProtKB/Swiss-Prot acceesion.

^5^Position in UniProtKB/Swiss-Prot protein entry.

^6^Whole exome sequencing.

### Variation statistics

After the SNVs are called, filtering procedures as described in materials and methods are used to identify high-quality SNVs. In order to investigate the distribution of the variants from 55 samples (some patients have more than one control or case) derived from 20 patients, we perform two types of comparison: 1) We compare with dbSNP to calculate the proportion of known and novel variants that we identify through our pipeline. 2) Within this study comparison is conducted by calculating the common and rare SNVs and the concordance (see descriptions of concordance, novel, common and rare SNVs in Methods) between cases and control sets. It is possible that sequencing errors can lead to identification of SNVs which in reality may not be present. Liu et al. [61] performed a comprehensive study where they showed that read preprocessing step did not improve the accuracy of variant calling but ability to flag duplication, local realignment and recalibration steps helped reduce false positive and also sequencing depth was important. The study also noticed SAMtools performed quite well in identifying SNVs. Nonetheless, validation of the novel nsSNVs identified through NGS analysis can be performed using traditional Sanger sequencing of PCR products. For example, novel variations found in this study if identified in the NCI-60 exome samples for breast cancer cell lines [62] can be easily validated using the procedure mentioned above. Further validation can also be performed using peptide mass-spectrometry [63] for the nsSNVs. Such validation will also become critical if any of the novel nsSNVs that is identified through this study is found in several samples and is hypothesized to be related to the disease.

#### ***Overlap with dbSNP and analysis of novel variations***

**Figure 3:**
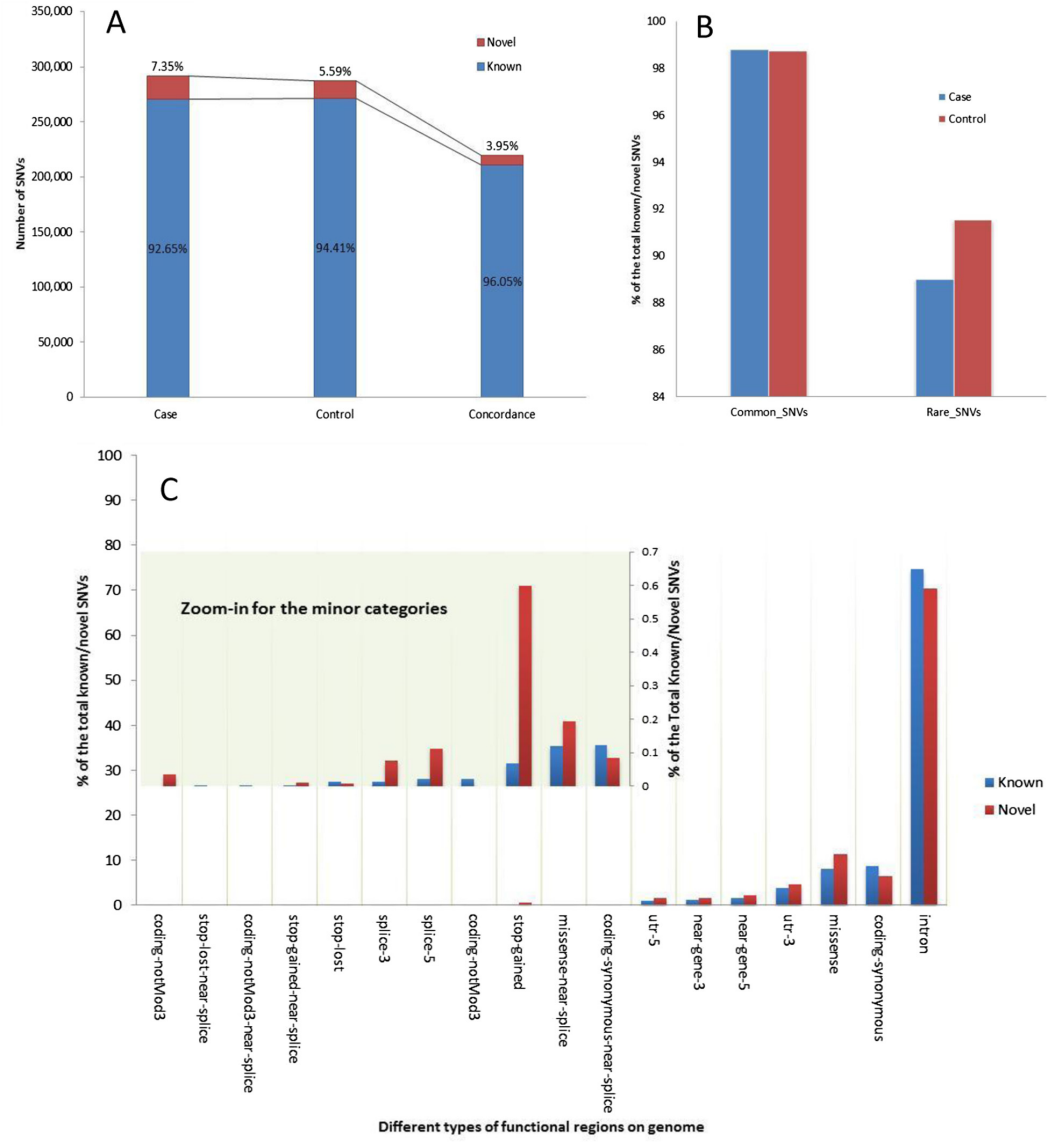
**Known and novel SNVs classification based on comparison with dbSNP. A)** The proportion of the novel/known SNVs in breast cancer cases and control. The last bar indicates SNVs that overlap in case and control. **B)** Distribution of common and rare SNVs in dbSNP. As expected SNVs that are rare (found in less than 10% of the samples analyzed) have a lower chance of being found in dbSNP. **C)** Functional annotation of novel and known SNVs. For functional groups that have lower numbers a zoomed-in view is shown.

Regarding concordance with dbSNP, individual samples show over 98% overlap with SNPs present in dbSNP version 137. In the total pool of the SNVs (all SNVs from the study), over 92% and 94% of SNVs in cases and controls respectively are shared with dbSNP (Figure 3A). The decrease in overlap percent when all the SNVs are pooled compared to what is seen in individual samples is due to overlap of dbSNP overlapping SNVs in multiple samples with the novel SNVs remaining mostly unique. More specifically, we find SNVs that are non-novel (also found in dbSNP) in multiple samples while novel SNVs found in individual samples are generally unique, thereby increasing the ratio of unique SNVs when all samples and cases are considered together (described in greater detail in the next section). If SNVs that overlap in both case and control are considered then the concordance with dbSNP increases to 96% as seen in the last bar of the graph in Figure 3A. This moderate increase is most likely because the mutations that overlap in case and control are most likely germline and are inherited and expected to be found in general population and hence in dbSNP.

The total pool of SNVs is further grouped by their calculated frequency among samples in this study. The SNVs with frequency higher than 10% are defined as common SNVs while the SNVs lower than this frequency are grouped into rare SNVs. The different percentage between novel and known (not present or present in dbSNP) SNVs is illustrated in Figure 3B. As shown, in both cases and control samples, the common SNVs are more likely to have higher (almost 99%) overlap with dbSNP, while rare SNVs, which are present in less than 10% of the samples, have around 90% overlap with dbSNP. It is indeed possible that some of the novel mutations identified can be cancer drivers as suggested in a recent paper by Khurana et al. [29].

In order to explore the distribution preference of SNVs of novel and known SNVs on genomic functional regions, all the SNVs were annotated using Seattleseq Annotation 137 web service [41]. Although not statistically significant, from our dataset it appears that novel SNVs are more prone to affect protein coding regions such as missense, stop-gain, and splicing (Figure 3C).

#### ***Distribution of SNVs in cases and controls***

**Figure 4:**
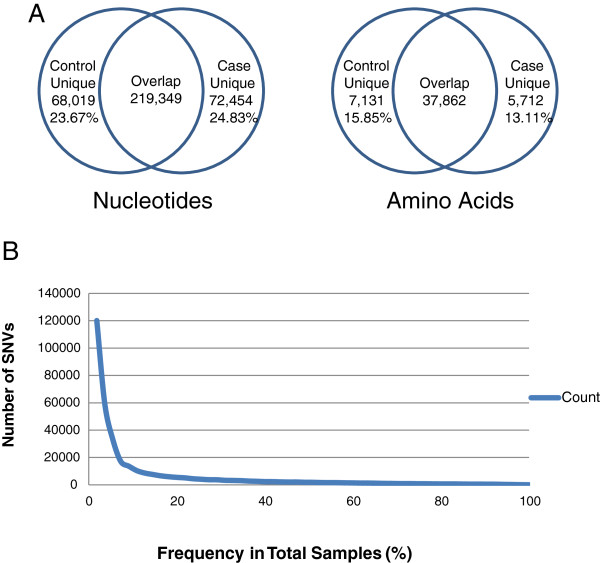
**SNV statistics. A)** Numbers show the total nucleotides and amino acids affected by SNVs. **B)** Visual representation of the distribution of SNVs in samples, showing that nearly 68% of all SNVs appear in 10% or lower frequency.

In terms of overlap of SNVs in cases and controls- we find that majority of the SNVs and nsSNVs overlap in case and control samples with ~ 20% of such unique variations found in the nucleotide level and ~15% in amino acids (Figure 4A). The correlation of SNVs between the different data sets was further analyzed using phylogenetic analysis methods described later in this manuscript. Comparing the number of SNVs that appear in one or more of the samples shows that the largest percentage of SNVs observed (33.34%) in the total pool of SNVs only appear in frequency of 1.18% (one sample). This distribution shows the heterogeneity of the SNVs in the samples and follows a trend similar to previous studies [29,64]. The graph (Figure 4B) presents a visual representation of the data, showing that nearly 68% of all SNVs in the data set appear in 10% or lower frequency of the total samples. In terms of novel variations for individuals they range from 1.27%-2.99% for control and 1.38%-4.00% for case. There is only once case sample (A7-A0DB-A272) with 15.72% novel SNVs. Based on the clinical data it is not clear why such a high number of SNVs are found in this particular case. In TCGA this same patient has another case sample where the numbers of novel SNVs are within the range observed for other patients. The associated phylogentic analysis (details in next section) provides an easy visualization and identification of samples that do not have a normal distribution of SNVs and hence can be followed up during the CSR curation process.

### Functional analysis

The methodology adopted for functional analysis is based on our earlier work [26,27]. In summary, we first evaluated the overall impact of the nsSNVs from the case and control samples on the entire proteome (proteome-wide analysis) in terms of effects on functional sites such as active sites, binding sites, co- and post-translational modification sites. Then we evaluate which domains and pathways are most affected by variation. Additionally, we also perform Gene Ontology, pathway and keyword analysis of the novel nsSNVs to better understand the effects of variations which are presumably rare.

#### ***Proteome-wide analysis of the effects of nsSNVs***

A broad analysis of all identified nsSNVs and also novel nsSNVs (variations not found in dbSNP and other variation databases) was undertaken get a comprehensive overview of the functional impact of variation. For this analysis all the nsSNVs derived from the CSR project are integrated into a proteome-wide analysis CSR companion tool SNVDis [28]. SNVDis is integrated into a High Performance Integrated Virtual Environment (HIVE) [30] that allows proteome-wide analysis of the nsSNVs. The SNVDis tool home page shows nine sources of variation data with two of them coming from this study (TCGA-Breast-Control and TCGA-Breast-Case). The default proteome that the analysis is performed on is UniProtKB/Swiss-Prot defined human proteome. In the analysis box in SNVDis one can choose what type of analysis they wish to perform. For example, selecting TCGA-Breast-Control and TCGA-Breast-Case and binding site will retrieve all nsSNVs that alter protein binding sites (as defined by UniProtKB/Swiss-Prot [20] and CDD [33] curators). This tool provides a comprehensive overview of how the nsSNVs affects active sites, binding sites, N-linked glycosylation sites, protein domains and pathways. For example, selecting the active sites (includes site annotations from both UniProt and CDD) that are affected by nsSNVs from the breast cancer case and control samples retrieves 56 sites in 44 proteins.

For pathway and domain analysis SNVDis estimates the number of expected variations to find in the domain or pathway based on uniform distribution of nsSNVs. For pathway analysis the UniProtKB/Swiss-Prot is selected and from the ‘Select dataset’ box TCGA-Breast-Control and TCGA-Breast-Cancer is selected and from the ‘nsSNV analysis on’ box ‘Pathways’ tab is selected followed by selected of the ‘by significance’ option. A p-value cutoff of 0.0000001 is chosen. The top five pathways that are affected when both cases and controls are taken together are Nicotine degradation (observed: 81, expected: 33.2, p value: 1.11E-16), FAS signaling pathway (observed: 81, expected: 33.2, p value: 1.11E-16), DPP-SCW signaling pathway (observed: 76, expected: 37.9, p value: 5.83E-10), Cadherin signaling pathway (observed: 433, expected: 280.0, p value: 3.93e-20) and Blood coagulation (observed: 156, expected: 87.2, p value: 1.69e-13). No immediate correlation to cancer pathways can be drawn from this analysis other than the fact that changes to signaling pathways are considered to be important in cancer progression [65].

Using a similar protocol as described above the top five functional domains (sorted based on p-value) that are affected in case and control samples are found to be almost identical: the transmembrane olfactory receptor (Pfam ID: PF13853), the cysteine rich domain that occurs alongside the TIL domain (PF12714), the glycoprotein-fucosylgalactoside a-N-acetylgalactosaminyltransferase domain (PF03414), a mammalian taste receptor protein domain (PF05296) and a protein kinase domain (PF00069) for the case sample and for the control samples instead of the kinase domain the glycoside hydrolase family 18 domain (PF00704) has over-representation of nsSNVs. Interestingly the greatest difference observed between control and sample analysis was the fact that the hyaluronan/mRNA binding domain (PF04774) is significantly affected more in the case samples (observed: 17; expected: 3.6; p value 1.26E-12) than the control samples (observed: 9; expected: 3; p value 6.41E-03). Increased levels of hyalunonan have already been correlated to breast cancer and often are used as a marker [66]. More samples would need to be analyzed to confirm this correlation.

As described earlier the number of case only or control only nsSNVs are less than 20% of the total variations and analyzing for over- or under-representation for cases and controls separately did not result in any appreciable differences in the highly affected pathways and domains. Therefore, a more detailed analysis of just the novel nsSNVs and their effects on functional sites was undertaken, results of which are described in the next section.

#### ***Enrichment analysis of novel nsSNV affected proteins***

In addition to the proteome-wide analysis of all nsSNVs an analysis of genes that are impacted by novel nsSNVs was also performed. For this analysis from case samples a total 17,177 novel nsSNVs containing gene accession numbers are mapped to 8,896 UniProtKB/Swiss-prot proteins and for novel nsSNVs in controls 13,523 gene identifiers are mapped to 6,961 UniProtKB/Swiss-Prot proteins. A decrease in the number of proteins compared to the number of initial RefSeq gene identifiers is because UniProtKB/Swiss-Prot entries represent the canonical protein whereas the genes in RefSeq can represent different isoforms. An initial analysis using the UniProtKB/Swiss-Prot keyword ‘Disease’ shows that the keyword is over-represented in the gene list having novel nsSNVs from case (observed: 1524; expected: 1211; p-value: 5.22E-21). Novel nsSNVs in the controls also appear to be over-represented albeit with a less significant p-value (observed: 1155; expected: 948; p-value: 6.46E-09). Based on UniProtKB/Swiss-Prot protein entry annotations of genes that are considered oncogenes, proto-oncogenes and tumor suppressors; for novel nsSNVs that are only found in case samples there seems to be a slight over-representation of tumor suppressor genes (observed: 43; expected: 27; p-value: 3.84E-03). It is important to note that cancer disease annotations in UniProtKB/Swiss-Prot (or in any other database) are far from being comprehensive. As more patient samples are analyzed and the disease specific annotations improve it will be possible to identify through this type of analysis if specific genes that are involved in cancer do indeed have higher level of mutations both in the controls and cases.

Additional functional analysis of genes which novel nsSNVs was performed to investigate if specific pathways, protein families or Gene Ontology (GO) terms are over- or under-represented. Pathway analysis shows that several of the top pathways (Table 2) are known to be involved in cancer. Other notable pathways known to be involved in cancer with less significant p-values include Wnt signaling pathway (observed: 205; expected: 161.67; p-value: 9.29E-02), Angiogenesis (observed: 119; expected: 87.71; p-value: 1.44E-01) and EGF receptor signaling pathway (observed: 86; expected: 64; p-value: 8.65E-01).

**Table 2:** Functional analysis of novel nsSNV containing genes

**Analysis**	**Functional object**	**Observed**	**Expected**	**+/-**	**P value**
PANTHER pathways	Integrin signaling pathway	147	99.09	+	6.43E-04
	Cadherin signaling pathway	109	74.91	+	2.15E-02
	Endothelin signaling pathway	62	38.88	+	6.55E-02
	Gonadotropin releasing hormone receptor pathway	174	133.70	+	7.71E-02
	Nicotinic acetylcholine receptor signaling pathway	75	49.78	+	8.78E-02
PANTHER protein classification	Cell adhesion molecule	443	316.70	+	9.28E-10
	G-protein modulator	346	238.47	+	3.82E-09
	enzyme modulator	906	730.11	+	5.69E-09
	kinase	431	312.91	+	1.28E-08
	cytoskeletal protein	585	452.77	+	1.02E-07
GO biological process	Cell communication	2333	2002.60	+	3.16E-14
	Cell adhesion	820	616.80	+	6.15E-14
	Signal transduction	2204	1905.41	+	5.32E-12
	Cellular component organization	773	595.00	+	4.72E-11
	Protein modification process	804	630.55	+	5.80E-10
GO molecular function	Hydrolase activity	1295	1053.92	+	1.81E-12
	Transferase activity	962	759.03	+	1.12E-11
	Protein binding	1715	1456.91	+	5.40E-11
	Transmembrane transporter activity	609	460.35	+	9.79E-10
	Enzyme regulator activity	732	576.51	+	1.05E-08
GO cellular component	Cytoskeleton	585	452.77	+	2.03E-08
	Intracellular	681	540.47	+	3.98E-08
	Actin cytoskeleton	302	229.94	+	8.66E-05
	MHC protein complex	18	40.30	-	2.37E-03
	Extracellular matrix	327	264.55	+	3.33E-03

Over- and under-representation of Gene Ontology (GO) terms, PANTHER pathways and protein classification in the list of genes which have novel nsSNVs provides an overview of what broad effects these novel variations might have. The major terms for Gene Ontology (GO) Biological Processes that are highly over-represented (p-value >1E-24) are metabolic process, cellular process and transport (primarily protein and ion transport). Table 2 provides a breakdown of the major terms. For GO Molecular Function the major terms include catalytic activity, binding (includes protein binding) and transporter activity (p-value >1E-9). Other notable GO Molecular Function includes kinase activity (observed: 432; expected: 314.80; p-value: 1.60E-08) and ion channel activity (observed: 242; expected: 186.80; p-value: 7.75E-03). Many of the pathways identified as over-represented in the gene list are associated with cancer [67,68]. For example, it is known that integrins and cadherins initiate signaling pathways that control the activity of Rho family GTPases [69]. Other terms that are over-represented are related to cell adhesion, cell communication, and signal transduction all of which are associated proteins that play active roles during tissue development and tumour metastasis [69]. Additional over-represented GO terms such as protein modification (e.g. post-translational modification (PTM) and enzymatic activity was further investigated to see if any of the mutations were resulting in loss of function PTM motif or active or binding sites of proteins.

**Figure 5:**
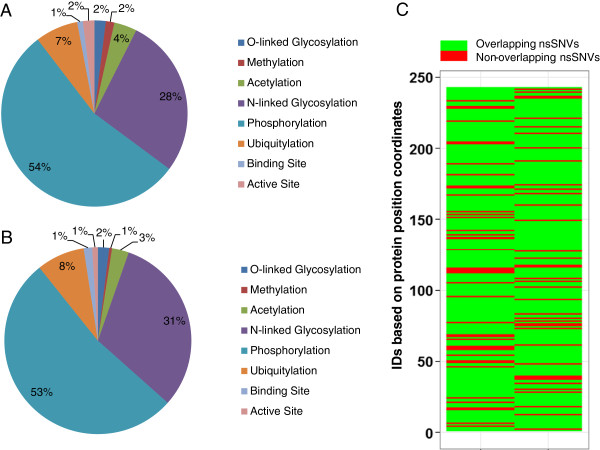
**Distribution of functional sites from UniProtKB/Swiss-Prot that affected by nsSNVs. A)** Distribution of loss of functional sites caused by nsSNVs from cases. **B)** Distribution of loss of functional sites caused by nsSNVs from controls. **C)** Heatmap representation of the overlap of nsSNVs in cases and control (nsSNVs found in both cases and controls are marked green and marked red if they are unique to case or control. All protein accessions and corresponding position were given a numerical ID (Additional file 1: Table S1) to facilitate visualization of the vertical axis in the heatmap.

*Functional analysis of proteins with variations in functional sites.* A more comprehensive analysis of the effects of nsSNVs is undertaken to better understand how they affect the functional sites. For this analysis all nsSNVs from case and control samples are mapped to UniProtKB PTM sequence features and dbPTM entries to analyze the distribution of nsSNVs on PTM sites. The database dbPTM is a non-redundant collection of experimentally verified PTM sites extracted from various related databases and publications [37]. dbPTM currently consists of 18 different types of PTMs from related databases and publications. Out of approximately 30,000 nsSNVs identified in case samples and similar numbers from controls the following functional sites were found to be affected: O-linked Glycosylation (case: 4; control: 4), Methylation (case: 3; control: 1), Acetylation (case: 8; control: 6), N-linked Glycosylation (case: 56; control: 64), Phosphorylation (case: 110; control: 108), Ubiquitylation (case: 15; control: 17), Binding Site (case: 2; control: 3), Active Site (case: 4; control: 2). Protein accession numbers and amino acid position for this data is provided in Additional file 1: Table S1 and graphically represented in Figure 5A and 5B. A search in UniProtKB/Swiss-Prot shows that several of these proteins that are affected by variation are implicated in cancer based on annotation in the Comments section of the entry (CC lines) [20]. Examples include, calcium-activated chloride channel regulator 2 (CLCA2), which is considered to act as a tumor suppressor in breast cancer [70]; ADAMTS-like protein 3 (ADAMTSL3) is expressed by malignant epithelial cells in colon cancer, as well as breast, prostate, renal and skin tumors [71]; clusterin is an anti-apoptotic protein associated with breast, prostate and other cancer types [72]; aberrant expression of macrophage colony-stimulating factor 1 (CSF1) can promote cancer cell proliferation, invasion and formation of metastases [73].

Based on our previous analysis results on the effects of variation on active sites of proteins and N-linked glycosylation (NLG) sites [26,27,29] we here provide additional details on these two important functional sites. Out of the four proteins whose active site is disrupted (protein arginine N-methyltransferase 6 (PRMT6), chymase (CMA1), kallikrein-5 (KLK5), sphingomyelin phosphodiesterase 3 (SMPD3)) two of the proteins have them disrupted in both cases and controls. PRMT6 active site variation is detected in 3 samples (one case and two controls from one patient (TCGA-BH-A0DK); CMA1 variation is detected in two case samples from same patient (TCGA-A7-A0DB-01A-11D-A272-09,TCGA-A7-A0DB-01C-02D-A272-09); KLK5 exists in 75% of the samples (16 case samples and 25 control samples); SMPD3 exists in only one patient sample (TCGA-A7-A0DB-01C-02D-A272-09). KLK5 and its trypsin-like serine protease paralogs have been associated with several cancers [74]. KLK5 active site variation which possibly deactivates the enzyme in a high percent of samples (both cases and controls) is most likely possible because one of the other paralogs (there are 15 members in the Kallikrein subfamily according to UniProtKB/Swiss-Prot) might have the ability to compensate for the loss of activity of one its members. For CMA1and SMPD3, the active site disruptions are found only in cases. Chymases are known to convert angiotensin I to angiotensin II, and influences of angiotensin I-converting enzyme gene polymorphisms on gastric cancer risks has been proposed before [75,76]. Finding this mutation in breast cancer tumor provides a novel target for further investigation on the role of this gene in breast cancer. Similarly, SMPD3 is known to catalyze the hydrolysis of sphingomyelin to form ceramide and phosphocholine and ceramide mediates cellular functions, such as apoptosis and growth arrest [20,77]. Mutations in SMPD3 have implicated the ceramide pathway in human leukemias [78]. Identification of loss of function mutation of SMPD3 due to a mutation in the active site of the enzyme in breast cancer tumor cells provides for the first time a potential association of this gene with breast cancer.

The asparagine-X-serine/threonine (NXS/T) motif, where X is any amino acid except proline, is the consensus motif for NLG. Therefore, mutations in this motif can lead to loss of NLG. Previously, we have shown through proteome-wide analysis how germline mutations can result in loss-of-glycosylation (LOG) [27]. In analyzing the somatic mutations of breast cancer patients we find 56 such LOG mutations in cases and 64 LOGs in controls (Additional file 1: Table S1). Out of these LOG sites 5 are unique to case samples (P08F94, Fibrocystin/PKHD1, position 830; P15151, Poliovirus receptor/PVR, position 122; P40126, L-dopachrome tautomerase/DCT, position 170; P52797, Ephrin-A3/EFNA3, position 102; P58170, Olfactory receptor 1D5/OR1D5, position 7; Q9NYQ6, Cadherin EGF LAG seven-pass G-type receptor 1/CELSR1, position 1289. All of these mutations appear to be novel except for Fibrocystin N- > S mutation at position 830 which amongst other mutations has been implicated in polycystic kidney disease [79,80] but not cancer.

### Phylogenetic analysis

It is our hypothesis that phylogenetic analysis of SNVs can provide a better way of characterizing control and case samples. SNV based characterization methods have been used before [81,82] but currently there are no phylogenetic analysis tools that allow for direct generation of trees from human SNVs identified in a next-generation sequence analysis pipeline. As described in Materials and Methods we have developed phyloSNP, which allows generation of SNV-shrunk genome alignments which can be used to create phylogenetic trees using existing tree building software such as MEGA [83], Clustal [39] etc.

**Figure 6:**
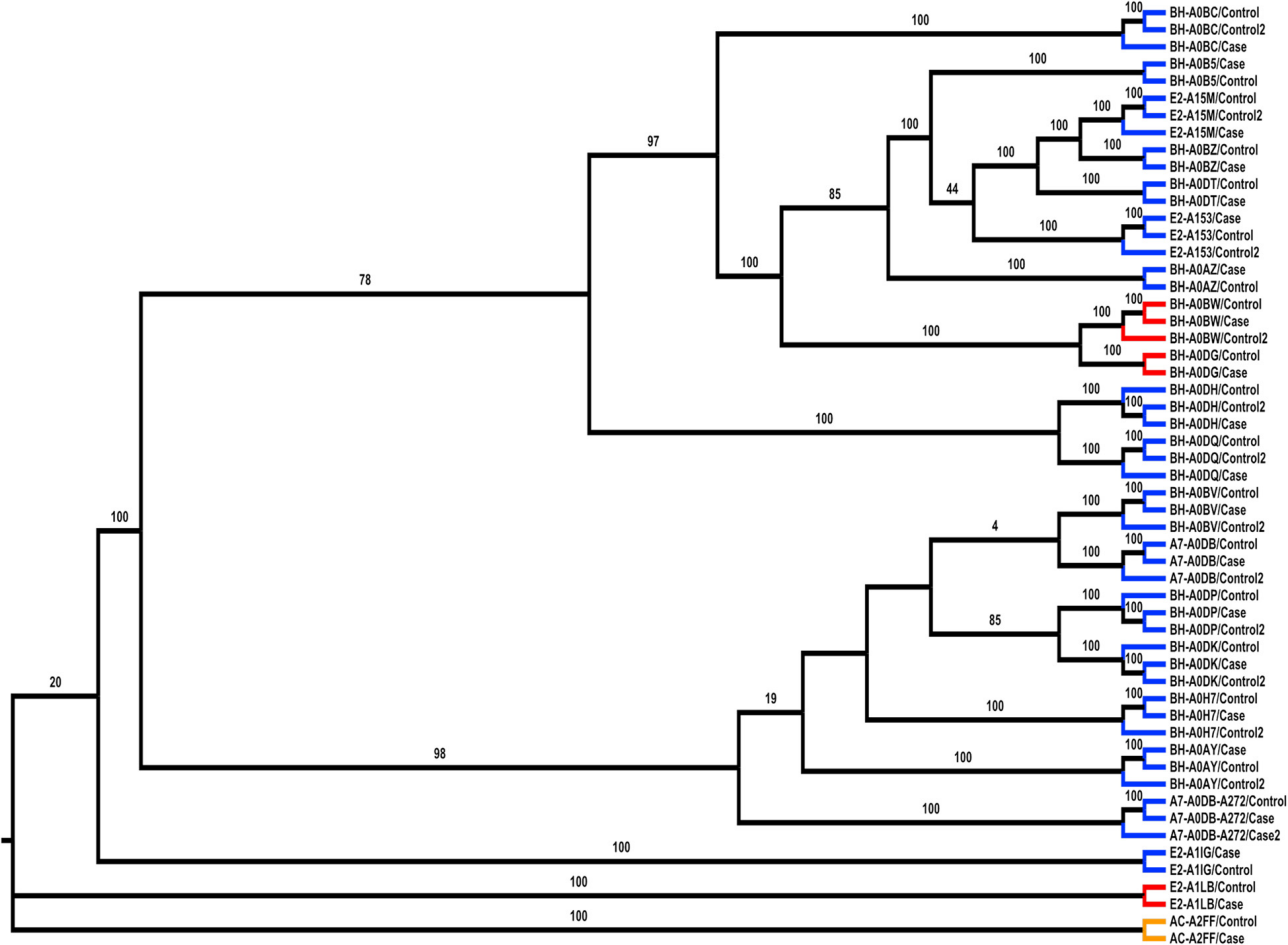
**Phylogenetic analysis of patient samples based on SNVs.** SNV-shrunk genome alignment comprising nucleotides from just the variation position is used (delta 0). Colored branches represent the ethnicity of the person from which the sample was taken with blue representing White, orange representing Asian, and red representing African-American. Bootstrap values of 75 and higher are shown. Two major groups are noticed with 98 and 78 bootstrap values.

Race and ethnicity information associated with patient samples may not always be the optimal method to categorize and evaluate genomic data [84]. Each sample is assigned an ID corresponding to the second and third TCGA call numbers along with a control or case tag. From here, all 55 samples are analyzed using phyloSNP shrunk-genomes with a delta 0 (only positions with the SNV was extracted and concatenated to create the SNV-shrunk genome). Increasing the delta to 10 did not significantly improve the tree (data not shown). A phylogenetic tree from the resulting genomic sequences was generated using ClustalW2 with 100 bootstrap replicates (Figure 6). All the cases and controls from the same patient branch together. For one patient (A7-A0DB) there are 6 samples (three cases and three controls). One case groups with two other controls from the same patient and the other two cases groups with another control from the same patient. It is interesting to note that although the numbers of novel SNVs are within 1.38%-4.00% for the sample A7-A0DB-A272 case2 there is 15.72% novel SNVs. For case1 from this patient the number of nsSNVs is within the accepted range. There is no information in the TCGA patient annotation files which provides any additional information as to why there is such high number of variations in this particular case. More samples need to be analyzed before any specific classification related correlations with disease or therapy can be made. It is evident even from the small sample size that SNV profiles are not always correlated with the ethnicity that was obtained from the sample annotations. It is our expectation that analysis of additional samples would allow us to create discrete bins which can then be used to categorize the samples, identify outgroups and even evaluate the quality of the sequence data. Such classification can be of great use for biomarker discovery processes if they can be correlated with disease outcome or other phenotypic characteristics. For 20 patient samples we did not find any correlation with the two major branches that can be seen in Figure 6 and clinical annotations available in the TCGA data files. It is important to note that the amount of clinical information available in the TCGA data files is limited and it is possible that with the availability of additional clinical or phenotypic data or through the analysis of data from additional patients we will be able to correlate classification results with specific clinical or phenotypic information.

### Web interface and usage

The CSR metadata home page (http://hive.biochemistry.gwu.edu/dna.cgi?cmd=csr) contains all available data related to this study. RefSeq gene and protein accession numbers can be used to retrieve results for single genes and SNV downloads are also available from the website. Variation results for single proteins are integrated with variation data from dbSNP, COSMIC and UniProt to easily identify variations that are sample specific and if they overlap with any known variations. Additionally, there are links from the sample pages to CBio cancer genomics portal [85] which provides additional information on the TCGA samples. Annotation data from TCGA data portal files are manually checked and combined with computationally analyzed mapping and SNV data and entered into CSR database. Clicking on the ‘Reviewed short read data’ brings the user to the ‘Curated SRA Browsing Interface’ which contains headers linked to ‘study’, ‘experiment’ and ‘sample’. Clicking on each of these links takes the user to the respective curated datasets which can be browsed. The study, experiment and samples are hierarchical. Whenever possible, identifiers are inherited from the primary database (in this case TCGA) to ensure easy tracking of data. For the experiments new identifiers were created to indicate if the samples are cases on control.

The described approach can be used by others by to analyze and document variations from other individuals available from TCGA and many other datasets available via dbGaP [82] thereby providing a better view of the human variome. It is expected that this type of bioinformatic analysis in conjunction with phenotypic information will allow researchers to correlate variation information with functional changes. Additional immediate impact of the CSR database will be providing access to variation data from individuals about specific genes. For example, a user studying the NM_001099771.2 gene (UniProt accession A5A3E0; POTE ankyrin domain family member F) might be interested in knowing what variations are present in this gene. A survey of variations for this gene in dbSNP shows that there are 186 known SNPs. From our analysis we find an additional 22 SNVs out of which there is one nsSNV which results in the loss of a phosphorylation site (amino acid position 918 Y|F mutation). The protein is known to be expressed in breast cancer cell lines [86]. In studies such as these if a rare nsSNV is observed in a critical functional site one would most likely try to estimate its impact through additional experimentation. An example of such follow up study where the variation impacts an active site of an enzyme could be assaying for buildup of substrate or decrease in the level of product [26]. If the nsSNV affects a PTM site [27] such as N-linked glycosylation then one would need to perform possibly additional analytical studies to evaluate the impact [87]. System level impact of nsSNVs and additional network analysis can also be applied to such analysis to better understand the personal genome [88-90]. For additional proteome-wide analysis of the impact of nsSNVs one can use SNVDis [28] as described in earlier sections.

### CloudBioLinux

CloudBiolinux [31] (http://www.cloudbiolinux.org), is a bioinformatics Virtual Machine (VM) that is implemented to run on Amazon EC2, on the open-source, private Cloud platform Eucalyptus (http://open.eucalyptus.com/), and on the desktop using VirtualBox (http://www.virtualbox.org). A VM is a fully-featured UNIX server, in a format of a single, downloadable binary file that executes on Clouds and desktop virtualization platforms. Cloud BioLinux includes a full Ubuntu Linux (http://www.ubuntu.com) and Galaxy Bioinformatics workbench interface [91], while users can start the VMs with a few clicks on the Amazon EC2 Cloud [92] without the need for any advanced technical knowledge. The Cloud BioLinux VM includes a suite of bioinformatic programming libraries for R, Perl, Ruby, and Python in addition to more than 100 pre-configured bioinformatics tools including BLAST, Glimmer, HMMER, PHYLIP, RasMol, Genespring, Clustalw, and the EMBOSS analysis suite. Given that the VM is accessible on the Amazon Cloud, it provides researchers with a large-scale, virtualized informatics infrastructure without the financial or time burden of owning and maintaining hardware and can therefore democratize access to computational resources for smaller laboratories which use NGS or other high throughput genomics technologies for biological experiments.

We leveraged the software libraries and tools that are available in the Cloud BioLinux VM, in order to install and run our pipeline with minimal effort as most dependencies were already available inside the VM. Furthermore, the VM format allows us to distribute the pipelines pre-configured and ready to execute in a single binary VM file that users can download and run on their desktop computers. This allows other researchers in the community to utilize our pipelines for their data analysis needs, without being required to spend time performing installations of bioinformatics tools or software libraries to configure the dependencies of the pipeline. The VM is available for download at (https://s3.amazonaws.com/cloudbiolinuxvms/cloudbiolinuxsra/cloudbiolinuxsra.ova) and users can boot it on their desktop following the instructions on the VirtualBox website (http://www.virtualbox.org/manual/ch01.html#ovf), while technical help is available through the Cloud BioLinux user group forum (https://groups.google.com/forum/?fromgroups#!forum/cloudbiolinux).

This workflow takes an SRA file and a reference sequence and calculates the coverage and SNVs of the reads when aligned against the reference sequence. The program is run from the command line using Perl and accepts as its parameters the base name and location of the reference index file, the name and location of the short read file; the name and location of the reference, and the output directory. The output will include a file containing putative SNPs, sequence coverage, and alignment statistics. The pipeline was run with a test input dataset from the NCBI-SRA (SRR052047.sra) and sample Bowtie2 indexes of the reference genome. The total run-time using a Cloud BioLinux Virtual Machine (VM, see Methods for more details) that use one CPU core out of four and two Gigabytes (GB) of memory on a laptop computer was approximately fifteen minutes.

## Conclusions

In this study we developed a workflow that involves identification and analysis of nsSNVs and curation of the metadata associated with TCGA samples. This information is available for browsing and downloads from CSR database which we plan to continuously update with representative datasets from all types of cancer with initial focus on curation of data from patients with both exome and RNA-sequencing data with matched cases and controls. We consider these datasets important and currently there are 615 such patients with all the data and sample types mentioned above. We plan to adhere to the evolving Human Variome Project recommendations and guidelines in terms of data formats and sharing. We also provide a CloudBiolinux and proteome-wide analysis platform to allow users to analyze NGS data in their research or biocuration pipelines. It is our belief that as more datasets are curated by our group and others, we will get a better understanding of the variability in human populations. One limitation of adopting the workflow proposed here is the ability of individual researchers to have the resources and expertise to perform next-generation sequence analysis. To overcome this limitation our group in collaboration with US Food and Drug Administration has been implementing the High-performance Integrated Virtual Environment (HIVE) which provides novel and known sequence read mapping and variation calling algorithms in a highly parallelized environment. It is our goal to provide both enterprise level HIVE for institutions and HIVE-in-a-box for individual users thereby democratizing the ability of scientists to work on Big Data.

### Availability

CSR: https://hive.biochemistry.gwu.edu/dna.cgi?cmd=csr

SNVDis: https://hive.biochemistry.gwu.edu/hive/snpdis.cgi?cmd=dmSnpdis.

CloudBioLinux: https://s3.amazonaws.com/cloudbiolinuxvms/cloudbiolinuxsra/cloudbiolinuxsra.ova.

## Abbreviations

CCDS: Consensus coding sequence; CSR: Curated short reads; GO: Gene ontology; TCGA: The cancer genome atlas; SNV: Single-nucleotide variation; nsSNV: Non-synonymous single-nucleotide variation.

## Competing interests

The authors declare that they have no competing interests.

## Authors’ contributions

RM conceived, designed and coordinated the study, developed a general outline for the algorithm and drafted the manuscript. CC developed the specific algorithm and was responsible for software design and implementation, and participated in the writing of the manuscript. JA, KKar and VS participated in the design and evaluation of the study and manuscript writing and algorithm design. KKra was responsible for implementing the workflow in Cloud BioLinux. WF participated in the phylogenetic and cluster analysis, MM and QW in biocuration, AG in database design and YP in comparative analysis. All authors read and approved the final manuscript.

## Supplementary Material

Additional file 1: Table S1 List of proteins with loss of functional sites due to nsSNVs in cancer case and control samples. Click here for file
